# Sex differences research is important!

**DOI:** 10.1186/s13293-025-00702-x

**Published:** 2025-03-10

**Authors:** Jill B. Becker, Sofia B. Ahmed

**Affiliations:** 1 Department of Psychology and Michigan Neuroscience Institute, University of Michigan, Ann Arbor, MI 48109 USA; 2 Editor in Chief, Biology of Sex Differences, New York, NY USA; 3 Faculty of Medicine and Dentistry, University of Alberta, Edmonton, Alberta Canada; 4 Women and Children’s Health Research Institute, University of Alberta, Edmonton, Alberta Canada; 5 President, Organization for the Study of Sex Differences, Bandera, Texas USA

On January 22, 2025, President Trump terminated ‘diversity, equity, and inclusion’ (DEI) discrimination in the federal workforce, and in federal contracting and spending”. Effective January 31, 2025, at 5 pm ET, all federal agencies were instructed to take down web pages that included ‘gender ideology extremism’. This meant all webpages on the National Institutes of Health’s (NIH) website that discussed sex as a biological variable (SABV) or how to implement the NIH’s SABV policy were rendered inaccessible. These actions shocked and dismayed scientists, who recognize that the study of sex differences is fundamental for science in order to improve the health of all humans, male and female.

SABV is necessary for scientific research to find rigorous, reliable, and reproducible answers for the causes and treatment of diseases. The sex of an individual profoundly impacts their health and susceptibility for disease. After decades of research that had focused mostly on male subjects in basic research and clinic studies, research needs to include females so that the findings will apply to the health of all individuals across the lifespan. A commentary in 2014 in the journal *Nature* by Francis Collins, the then Director of the NIH, and Janine Clayton, The Director of the Office of Research on Women’s Health (ORWH), indicated that “inadequate inclusion of female cells and animals in experiments and inadequate analysis of data by sex may well contribute to the troubling rise of irreproducibility in preclinical biomedical research” [1].

Effective January 25, 2016, the National Institutes of Health (NIH) instructed all researchers funded by the NIH who were studying vertebrate animals or humans to include both females and males in their studies. Results of their studies were to be analyzed by sex and the results reported with the sex of the subjects included among the data. To our dismay, the NIH websites that explain SABV, and NIH websites that provided resources for conducting SABV research, were no longer accessible for a period of time. As of this writing on February 26, 2025 the SABV statement on the ORWH website has now been reinstated. The resources for conducting SABV research, that were previously available, are still met with “page not found”. The webpage describing the “Specialized Centers of Research Excellence (SCORE) on Sex Differences” program, a signature program of ORWH, are now labeled “Historic document published prior to January 20,2025”. Of note, Biology of Sex Differences has a collection of articles on “Sex/Gender Differences in Social Determinants of Health at Specialized Centers of Research Excellence (SCORE) on Sex Differences**”.**

As a clinician-scientist, Dr. Ahmed has devoted her career to the study of how sex and gender factors impact health. Sex refers to factors including genetics, anatomy, reproductive organs; gender is not only composed of identity, but also roles and relations which can directly impact health outcomes. For example, individuals who have more roles commonly ascribed to women (e.g., childcare, responsibility for household activities) are more likely to present to hospital with a recurrent acute coronary syndrome, irrespective of sex [2]. Studying biologically-based sex differences and how different exposures, roles and behaviors contribute to health outcomes is fundamental to improving everyone’s health at all life stages. This is not ‘radical DEI implementation’ or ‘gender ideology extremism’, it is just common sense. A precision health approach informed by research is required to provide “the right treatment to the right person at the right time”. Understanding how sex- and gender-specific factors across the life cycle impact health is fundamental to providing the best care and ultimately optimizing health outcomes for all.

As the Editor in Chief of the journal *Biology of Sex Differences*, Dr. Becker sees many scientific reports of how females and males differ. For example, there were sex differences in mortality due to Covid-19 - more men died than women [3, 4], at least partly because of sex differences in the immune response. Most people don’t realize it, but more women die of heart disease than men, yet more men are diagnosed with heart disease [5, 6] due to a lack of understanding of sex differences in cardiovascular disease and its risk factors. In Alzheimer’s Disease more women than men have the disease and we still do not understand the fundamental causes of the disease [7–10], nor is there an effective treatment for the disease, perhaps because most of the studies to date have been conducted with only male animals [11].

Sex differences research is not “woke gender ideology.” Sex differences research is fundamental biological science. The sex of an individual is determined by the genes on the sex chromosomes (XX is female, XY is male) and the hormones produced by the developing gonads acting on the body during development and after puberty. People differ in the sexual characteristics they develop due to environmental influences and biological differences. Furthermore, individuals that experience stress during development in the uterus or as infants have greater likelihood of mental health disorders later in life and there are sex differences in how this happens and what the outcomes are [12–14].

For example, variation in the number of X or Y chromosomes (e.g., XYY or XXY) influences total brain volume in humans and other species [15, 16]. Variation in genes that affect the development of the gonads can result in an XY individual that develops without testes and has female characteristics. Failure to synthesize gonadal hormones during development, making too much androgen due to an inherited genetic trait, or not having receptors to detect androgen, all affect the development of the body so that the apparent sex may not be consistent with the sex chromosomes. To use common language, this means that biological variation results in more than two forms; it is not as simple as male and female.

The idea that by wiping clean or diminishing the evidence of SABV or DEI from the websites of the US government it is somehow going to remove the reality of biological sex differences among individuals is wrong. The executive order called *“Defending women from gender ideology extremism and restoring biological truth to the federal government*,*”* indicates that the government will only “recognize two sexes, male and female.” Substituting opinion for scientific fact does not make it so. We call on the NIH to restore all of the resources to help scientists conduct sex differences research in a reliable way as a part of the rigor and reproducibility criteria for NIH grants.

The executive order went on to say, “Federal funds shall not be used to promote gender ideology. Each agency shall assess grant conditions and grantee preferences and ensure grant funds do not promote gender ideology”. If this executive order, and other directives from the president, result in the failure to fund research that investigates sex differences, the US will fall even farther behind in our science and health care. US life expectancy has declined in the last two decades, falling further behind that of most other peer wealthy nations [17–20]. The urgency of understanding factors, and particularly sex-specific factors, contributing to Americans’ diminishing lifespans cannot be overstated. The executive order is completely unacceptable.

As a professor, Dr. Becker teaches undergraduates. In her class on Hormones and Behavior, students are shocked to learn that the drugs they take have been found to be effective in males, but we don’t know if they work in women. It’s not that women are not included in the studies, but the clinical trials don’t usually have enough female subjects to be able to tell if the drug worked in the women. Furthermore, the Food and Drug Administration (FDA) does not require that the results of clinical trials be made available to the medical community with the data disaggregated by the sex of the subjects. This means that scientists are not able to analyze that data to find out if the drugs, that are approved for use in all, actually work in both men and women. A 2001 U.S. Government Accountability Office report outlined that 8 of 10 drugs withdrawn from the U.S. market over a four year period (1997–2000) posed greater risk to women than men [21]. It was concluded this was due to a lack of female representation in the research pipeline leading to FDA approval.

We need biomedical research that investigates sex differences in neurological disorders, immunology, cardiovascular disease and function, obesity and metabolism, addiction and motivation, mental health, bone health and function, cancer, aging – the list goes on and on. We know too little to stop now and what we know indicates we help everyone when we study sex differences.
